# Polymeric Coating of Silica Microspheres for Biological Applications: Suppression of Non-Specific Binding and Functionalization with Biomolecules

**DOI:** 10.3390/polym14040730

**Published:** 2022-02-14

**Authors:** Dario Brambilla, Alessandro Mussida, Anna M. Ferretti, Laura Sola, Francesco Damin, Marcella Chiari

**Affiliations:** Institute of Chemical Science and Technology “G. Natta”, National Research Council of Italy (CNR-SCITEC), 20131 Milan, Italy; alessandro.mussida@scitec.cnr.it (A.M.); anna.ferretti@scitec.cnr.it (A.M.F.); laura.sola@scitec.cnr.it (L.S.); francesco.damin@scitec.cnr.it (F.D.); marcella.chiari@scitec.cnr.it (M.C.)

**Keywords:** silica, microparticles, polymer, coating, biosensing, biomolecules, DNA, protein, streptavidin

## Abstract

The use of micro- and nanoparticles in biological applications has dramatically grown during the last few decades due to the ease of protocols development and compatibility with microfluidics devices. Particles can be composed by different materials, i.e., polymers, inorganic dielectrics, and metals. Among them, silica is a suitable material for the development of biosensing applications. Depending on their final application, the surface properties of particles, including silica, are tailored by means of chemical modification or polymeric coating. The latter strategy represents a powerful tool to create a hydrophilic environment that enables the functionalization of particles with biomolecules and the further interaction with analytes. Here, the use of MCP-6, a dimethylacrylamide (DMA)-based ter-copolymer, to coat silica microspheres is presented. MCP-6 offers unprecedented ease of coating, imparting silica particles a hydrophilic coating with antifouling properties that is able to provide high-density immobilization of biological probes.

## 1. Introduction

Microparticles composed of different materials, i.e., polymers, inorganic dielectrics, and metals, are widely explored for varying biomedical applications [1,2,3]. Their use in immunoassays has dramatically grown during the last few decades due to the ease of protocol development and compatibility with microfluidics devices [4]. Microsphere-based diagnostic tests and assays are usually based upon the specific interaction of an antigen (Ag) and antibody (Ab). Sub-micron-sized microspheres of different materials are used as the solid support; Ab or Ag can be adsorbed or covalently bound onto them. These “functional” microspheres, when mixed with a sample containing the opposite reactant, capture the target of interest that can either be detected or isolated [5]. Typically, an antibody, specific for one epitope on the antigen, is immobilized on particles such that the antigen recognition sites (Fab) region of the IgG molecule is oriented away from the particle surface. Upon incubation with the particles, the analyte of interest binds to the antibody on the particle surface. A second labeled antibody, specific for another epitope on the antigen, also links to the antigen, forming a sandwich [6]. Micro/nanoparticles can be used to separate specific biomarkers by immune isolation from complex mixtures. The use of magnetic microparticles as the solid phase has revolutionized the field of clinical chemistry by facilitating the development of more sensitive high-throughput automated immunoassays [7]. An example of such an application is the isolation of extracellular vesicles (EVs) from plasma. Several specific antigens on the surface of EVs, such as CD63, CD81, CD82, CD9, Alix, annexin, EpCAM, and Rab5, are targeted by micro/nanoparticles functionalized with antibodies against these surface markers. For example, CD34 is a unique biomarker of acute myeloid leukemia (AML) blasts [8]. Antibodies against CD34 can be immobilized on magnetic microbeads to purify EVs from the plasma of AML patients by means of an immunoaffinity interaction between CD34 and their antibodies. This technology is also applied to isolate and purify extracellular vesicles derived from a particular cell type, such as tumor cells [9]. Controlling the surface characteristics of particles is crucial in many types of biological applications involving micro/nanoparticles since they impact the interactions between particles and biopolymers (e.g., protein, nucleic acids) and, thus, the overall performance of the system [10,11]. In fact, by tuning the surface properties of a microsphere, it is possible to influence its loading capacity, binding kinetics, and non-specific binding [12]. Different types of nanomaterials exhibit functional groups on their surface that are used in the first steps of functionalization. Generally, homo- or hetero-bifunctional cross-linkers are used to bind biological molecules. For silica NPs, the most used linkers are aminosilanes that introduce an amino group on the NPs’ surface for the next bio-conjugation [13]. A number of properties, including dispersibility, biocompatibility, and mechanical stability, can be conveniently altered by applying surface coatings to nanoparticles. An effective way to modulate surface properties is by utilizing polymeric coatings. Polymeric coatings represent a powerful tool to control and optimize the surface characteristics of a particle. The presence of a hydrophilic polymeric layer between the particle and a biomolecule can improve colloidal stability, reduce nonspecific interaction, and confer peculiar properties to the particles [4]. This feature is an important aspect when working with complex biological samples. Using polymeric coatings, it is also possible to introduce reactive moieties that allow to immobilize biological probes on the particle surface. Here, the use of MCP-6, a dimethylacrylamide (DMA)-based ter-copolymer, to coat silica microspheres is presented. Our group has developed a family of copolymers whose progenitor is copoly(DMA-NAS-MAPS), a copolymer obtained by the radical copolymerization of DMA, N-acryloyloxysuccinimide (NAS), and 3-trimethoxysilyl)propyl methacrylate (MAPS) [14]. NAS is the reactive moiety of the copolymer and provides biological probe immobilization through an amine coupling reaction [15]. A number of polymers derive from this parent polymer. They form a uniform and ultrathin coating of different materials through a double mechanism that involves both adsorption and covalent grafting. In fact, DMA, the monomer that forms the backbone of this polymer family, has intrinsic self-adsorption properties obtained by a combination of hydrogen bonds and Van der Waals forces [16], while the condensation of MAPS to surface-exposed hydroxyl groups provides further covalent stabilization. A fast, robust, and reliable coating procedure provides a hydrophilic surface where biological probes (both proteins and nucleic acids), immobilized with high density, remain accessible and retain an active conformation. In addition, the antifouling character of the coating contributes to its success in numerous analytical applications, especially those related to microarray technology [14,17,18,19]. By the post-polymerization modification of copoly(DMA-NAS-MAPS), azide groups were introduced through a reaction of NAS with 3-azido-1-propylamine. The polymer is called MCP-6. This functional moiety takes part in copper-catalyzed and strain-promoted azide-alkyne cycloaddition reactions (CuAAC and SPAAC, respectively), allowing the high density immobilization of alkynyl modified biomolecules [20]. In the present work, we demonstrate that MCP-6 can be successfully used to coat silica microspheres, retaining its peculiar properties. The coating procedure is fast, reliable, and does not require expensive or sophisticated instrumentation. Coated silica microparticles are functionalized with common biological probes, including DNA, streptavidin, and antibodies, thus enabling the easy and reliable fabrication of silica supports for biological assays with tailored properties.

## 2. Materials and Methods

### 2.1. Materials

Ammonium sulfate ((NH_4_)_2_SO_4_), dibenzocyclooctyne-N-hydroxysuccinimidyl ester (DBCO-NHS ester), phosphate buffer saline tablets (PBS), streptavidin, 3-azide-1-propylamine, bovine serum albumin (BSA), sodium dodecyl sulphate (SDS), Tris, HCl, Amicon Ultra 30 MWCO and 100 MWCO centrifugal filters, dimethylacrylamide (DMA), N-succinimidyl acrylate (NAS), 3-(trimethoxysilyl)propyl methacrylate (MAPS), and polyclonal rabbit IgG were purchased from Sigma-Aldrich (St. Louis, MO, USA). The polymer MCP-2 was purchased from Lucidant Polymer (Sunnycale, CA, USA). Bradford Protein Assay Dye Concentrate was purchased from Bio-Rad (Hercules, CA, USA). Goat antirabbit IgG was purchased from Jackson ImmunoResearch (Baltimore, PA, USA). Oligonucleotides COCU8: 5′-GCCCACCTATAAGGTAAAAGTGA-3′, COCU11: 5′-TCACTTTTACCTTATAGGTGGGC-3′, and DNA-negative: 5′-ACTTAGGACTCAGTACAGGATAGACTTGATATCGGTTGGA 3′ were synthesized by MWG-Biotech AG (Ebevsberg, Germany). COCU8 was modified either with DBCO-linker or biotin at 5′ end, COCU11 was labeled with Cy5 at 5′ end, and DNA-negative was labeled with biotin at 5′ end. Oligonucleotides were freeze-dried and resuspended in de-ionized water (DI water) at a final concentration of 100 μM before use. Silica microbeads were purchased from Bangs Laboratories Inc. (Fishers, IN, USA). Spectrofluorimetric analysis was performed using a Jasco FP-550 spectrofluorometer equipped with thermo-stated Peltier cell holder. Bradford protein assays were performed using a Thermo Labsystems Multiskan Ascent microplate spectrophotometer. ζ-Potential measurements were performed on a Zetasizer Nano ZS Instrument (Malvern Instruments Corp., Malvern, UK), and samples were loaded in a Zetasizer nano series Dip Cell Kit (Malvern Instruments Corp., Malvern, UK).

### 2.2. Synthesis of MCP-6

MCP-2 was modified by reaction with 3-azido-1-propylamine, as reported elsewhere [20]. To introduce the azido groups, a 20% *w*/*v* solution of the copolymer was prepared by dissolving it in dry THF, and a 2.5 molar excess with respect to the moles of NAS of 3-azido-propylamine was added to the crude material, assuming that the concentration of NAS along the polymer chain is 40 mM. The mixture was stirred for 5 h at room temperature and then diluted 1:1 with anhydrous THF. The polymers were precipitated in petroleum ether (10 times the volume of the reaction mixture), filtered on a Büchner funnel, and dried under vacuum at room temperature.

### 2.3. Coating of Silica Microspheres Using MCP-6

Silica microspheres (10% *w*/*v*, diameter = 1 µm) were sonicated in a water bath for 10 min and vortexed 30 s to ensure proper resuspension. Then, 50 µL (=5 mg) were transferred into a 1.5 mL tube and washed twice with MQ water. After each washing or incubation step, beads were separated from supernatant by centrifugation. Beads were resuspended in 1 mL solution of MCP-6 (1% *w*/*v* in 0.9 M ammonium sulphate) and incubated 30 min at 25 °C under stirring followed by 30 min at 25 °C without stirring. Beads were washed twice with 1 mL of MQ water and used for further experiments.

### 2.4. Zeta Potential Measurement

ζ-potential measurements were carried out at a wavelength of 633 nm with a solid state He–Ne laser at a scattering angle of 173° at 298 K on diluted samples (0.01–0.1 mg/mL particles) at pH 7. Each result was averaged from at least three measurements.

### 2.5. Antifouling Properties Evaluation

Twenty mg of silica microspheres was coated with MCP-6 as described in Section 2.3. Beads were washed with 1 mL of PBS and then incubated overnight at 37 °C under stirring with 1 mL of a 50 mg/mL protein solution (BSA or lysozyme) in PBS. Beads were washed three times with PBS. Beads were then resuspended in 150 µL of 0.1% SDS, incubated 10 min at 95 °C, and, after centrifugation, the supernatant was recovered. The step with SDS was repeated two additional times, and all supernatants were pooled and concentrated on an Amicon Ultra 3 MWCO centrifugal filter (10 min at 12,200× *g*) to a final volume of around 50 µL. The same procedure was repeated on 20 mg of uncoated silica microspheres as negative control. Samples were diluted five times using water, and the concentration of BSA or lysozyme released by beads upon SDS-mediated denaturation was assessed by Bradford protein assay.

### 2.6. Immobilization of Oligonucleotides on MCP-6 Coated Silica Microspheres

#### 2.6.1. Immobilization of Oligonucleotides

Five mg of MCP-6 coated silica microspheres was washed in 1 mL of PBS and resuspended in 100 µL of DBCO-modified COCU8 in PBS (different concentrations ranging from 1 to 20 µM were tested) and incubated overnight at 37 °C under stirring. Beads were washed twice with 1 mL of water and once with 1 mL of PBS.

#### 2.6.2. Hybridization with Complementary DNA

Five mg of beads functionalized with COCU8 was resuspended in 100 µL of Cy5-labeled COCU11 in PBS (at the same concentration used for COCU8 during immobilization step) and incubated for 1 h at 25 °C under stirring. Beads were centrifuged and supernatant was recollected. Beads were washed twice with 100 µL of PBS; after, beads were centrifuged and supernatant recollected. Supernatants were pooled together and, only in samples where the concentration of DNA used during incubation was 5 µM or higher, diluted 1:10 using PBS. Further, 150 µL of pooled supernatants (diluted or not) was mixed with 350 µL of PBS, and the fluorescence emission intensity at 658 nm was measured using a Jasco FP-550 spectrofluorometer in 1 cm quartz cuvettes.

### 2.7. Immobilization of Streptavidin on MCP-6 Coated Silica Microspheres

#### 2.7.1. Synthesis of DBCO-Modified Streptavidin

To 1 mL of 1 mg/mL streptavidin in PBS, 9 µL of 4 mM DBCO-NHS ester were added (6.67 equivalents). The solution was allowed to react 30 min at room temperature. Reaction was quenched adding 100 µL of Tris-HCl 1 M pH 8. After 5 min at room temperature, the solution was transferred to Amicon Ultra 30 MWCO centrifugal filters and the excess of DBCO-NHS ester was removed by centrifugation. The final volume was adjusted to 1 mL by adding PBS.

#### 2.7.2. Streptavidin Immobilization

Ten mg of MCP-6 coated silica microspheres was resuspended in 500 µL of 1 mg/mL DBCO-modified streptavidin and incubated overnight at 37 °C under stirring. Beads were then washed 3 times with 1 mL of PBS and finally resuspended in 100 µL of PBS.

#### 2.7.3. Capture of Biotinylated Oligonucleotides

One mg of streptavidin-coated silica microspheres, prepared as described in Section 2.7.2, was resuspended in 200 µL of 3 µM biotinylated COCU8 in PBS for 30 min at 25 °C under stirring. Beads were washed twice with 1 mL of MQ water and once with 1 mL of PBS. As a negative control, 1 mg of streptavidin-coated silica microspheres was used to immobilize biotinylated negative-DNA following the same experimental procedure. Both the aliquots were resuspended in 100 µL of 6 µM Cy5-labeled COCU11 in PBS and incubated for 1 h at 25 °C under stirring; then, beads were centrifuged and supernatant was recollected. Beads were washed twice with 100 µL of PBS, and, after each step, supernatants were recollected. Pooled supernatants were diluted 1:10 using PBS, and 150 µL of the diluted solution was added to 350 µL of PBS, and the fluorescence emission intensity at 658 nm was measured using a Jasco FP-550 spectrofluorometer in 1 cm quartz cuvettes.

### 2.8. Immobilization of Antibodies on MCP-6 Coated Silica Microspheres

#### 2.8.1. Synthesis of DBCO-Modified Rabbit IgG

To 500 µL of 1 mg/mL rabbit IgG in PBS, 12.3 µL of 4 mM DBCO-NHS ester was added (15 equivalents). The solution was allowed to react 30 min at room temperature. Reaction was quenched adding 50 µL of Tris-HCl 1 M pH 8. After 5 min at room temperature, the solution was transferred to Amicon Ultra 100 MWCO centrifugal filters and the excess of DBCO-NHS ester was removed by centrifugation. The final volume was adjusted to 500 µL by adding PBS.

#### 2.8.2. Rabbit IgG Immobilization

Ten mg of MCP-6 coated silica microspheres was resuspended in 500 µL of 1 mg/mL DBCO-modified rabbit IgG and incubated overnight at 37 °C under stirring. Beads were then washed 3 times with 1 mL of PBS and finally resuspended in 100 µL of PBS. As a negative control, 10 mg of MCP-6 coated silica microspheres was resuspended in PBS and followed the same experimental procedure.

#### 2.8.3. Interaction with Secondary Antibody

The two aliquots of beads prepared as described in Section 2.8.2 were resuspended in 40 µL of 0.5 mg/mL goat antirabbit IgG in PBS and incubated for 1 h at 25 °C under stirring. After centrifugation, the supernatant was recollected. Beads were washed once with 40 µL of PBS and supernatant was recollected. Supernatants were pooled and goat antirabbit IgG concentration was measured by Bradford protein assay.

### 2.9. Transmission Electron Microscopy

Transmission electron microscopy (TEM) images were taken by a ZEISS LIBRA 200 FE microscope that has a FEG source (200 kV of emission power) and is equipped with a second-generation column Ω filter. The microparticle size was measured by TEM Imaging Platform Olympus.

## 3. Results & Discussion

**Figure 1:**
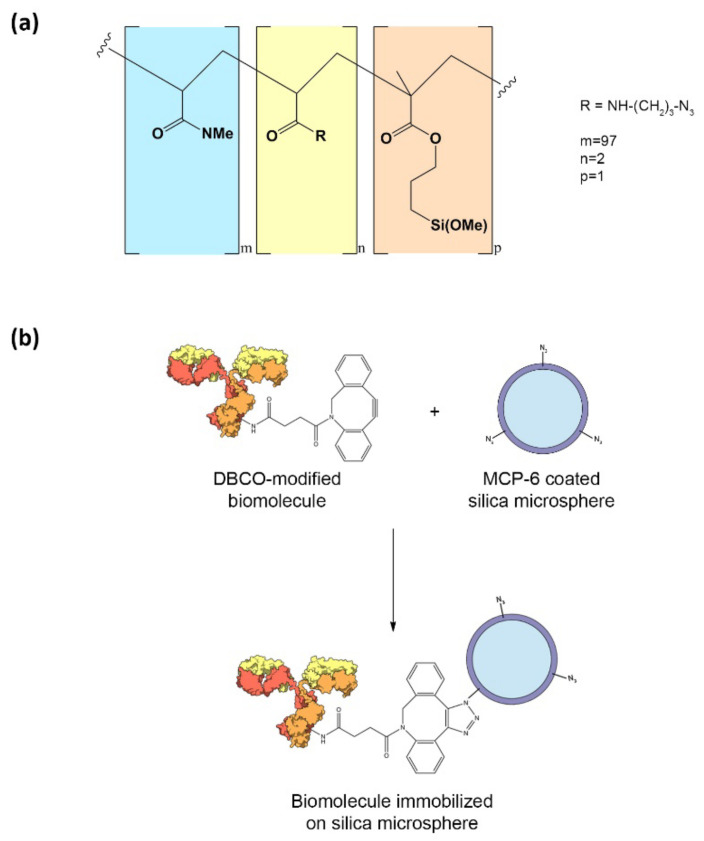
(**a**) Chemical structure of MCP-6; (**b**) schematic representation of immobilization of biomolecules via SPAAC: DBCO-modified biomolecule reacts with azide groups exposed on MCP-6 coated silica microspheres, forming a stable triazole that anchors the biomolecule on the particle.

When coating silica microspheres, choosing an appropriate polymer is crucial to determine the properties of the particles. The antifouling properties of the coating and biomolecule immobilization density are strongly influenced by surface chemistry. MCP-6, whose structure is shown in Figure 1a, satisfies both requirements as its DMA-based backbone maintains the antifouling properties of the progenitor polymer copoly(DMA-NAS-MAPS, while the azide functionality allows the immobilization of the biomolecules via strain-promoted azide-alkyne cycloaddition (SPAAC) reaction. The active esters used to immobilize the biomolecules via amine coupling suffer from hydrolysis in aqueous media [21]. This phenomenon leads to poor reproducibility and low immobilization yields, thus impairing the performance of downstream applications [22]. On the contrary, SPAAC reaction is not affected by the presence of water [23]. This “click reaction” occurs spontaneously without catalysts and represents a powerful tool to achieve an effective and reproducible immobilization of biomolecules on silica microspheres (Figure 1b).

**Figure 2:**
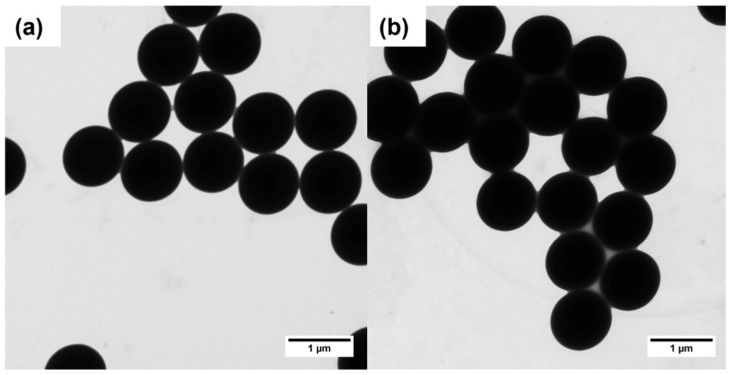
TEM images of (**a**) uncoated silica microspheres; (**b**) MCP-6 coated silica microspheres.

MCP-6 has proven to be highly efficient in coating glass and silicon microarray chips. The polymer interacts with these materials through both covalent (i.e., condensation of surface silanols with MAPS monomer) and non-covalent interactions (e.g., H-bonds and Van der Waals forces). The polymer is capable of coating silica beads that expose free silanols on their surface. To obtain a stable coating, the silica microbeads were immersed into a 0.9 M ammonium sulfate solution of the polymer (1% *w*/*v*). The salt acts like a salting out agent that, by limiting the polymer’s solubility in water, promotes its adsorption on the surface of the beads [24]. After 1 h of incubation, the silica beads were recovered by centrifugation and washed twice with water to retrieve the MCP-6-coated particles. A TEM analysis was performed to demonstrate that the dimension, shape, and dispersion of the silica microparticles were not affected by the polymer coating. The results, shown in Figure 2, confirm that, upon polymer adhesion on the microparticles, the morphology of the silica microspheres is not affected and there are no signs of aggregation.

The presence of the polymeric coating was demonstrated by zeta potential measurement. The uncoated and MCP-6 coated silica microbeads were dispersed and diluted in a NaCl solution and analyzed. The measured zeta potentials were −72 mV and −12.1 mV for the uncoated and MCP-6 coated beads, respectively. This result confirmed the successful formation of the coating. In fact, while bare silica presents a high density of negative charges on its surface, the coating masks these charges. The high difference between the zeta potential for the uncoated and coated beads (around 60 mV) suggests the presence of a uniform film on the beads. During the last 20 years, our group has acquired extensive knowledge on a family of DMA-based copolymers that includes MCP-6 [14,20,25]. All these copolymers, which originate from MCP-2, share similar behavior on solid surfaces. In a previous work [26], we measured the polymer thickness (10–15 nm), mass (about 200 ng/cm^2^), and density (0.18 g/cm^3^). Since the azide molar fraction is 2%, and its molecular weight is 100.12 g/mol, we can estimate the density of the azido groups on the surface, which is around 0.04 nmol/cm^2^.

**Figure 3:**
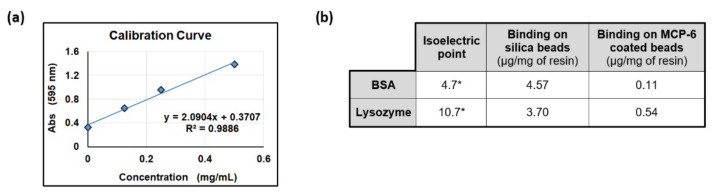
Protein quantification by Bradford protein assay. (**a**) Calibration curve; (**b**) isoelectric points and concentration of protein released from coated or uncoated beads after SDS treatment. * Isoelectric points for BSA and lysozyme are reported in the literature [27,28].

Subsequently, to demonstrate that MCP-6, once adsorbed onto silica beads, retains its peculiar antifouling behavior, the coated beads were incubated overnight with 50 mg/mL protein solution in PBS at 37 °C. In particular, bovine serum albumin (BSA) and lysozyme were chosen because of their tendency to interact non-specifically with surfaces. Furthermore, BSA and lysozyme possess different isoelectric points (4.7 and 10.7, respectively [27,28]); thus, the evaluation of their adsorption provides information on the role of the charges on the interaction with the surface. The uncoated silica beads were the negative control in this experiment. After incubation with proteins, the beads were washed with 0.1% SDS for 10 min at 95 °C in order to release the adsorbed albumin or lysozyme. The protein concentration in the diluted supernatant was measured with a Bradford protein assay. The results are shown in Figure 3.

As highlighted in Figure 3, MCP-6-coated and uncoated silica beads behave differently: uncoated beads release a high amount of protein after treatment with SDS, confirming the literature evidence supporting a strong non-specific adsorption of proteins onto silica surfaces [29,30]. Contrarily, MCP-6 coating confers antifouling properties to silica beads since only small traces of proteins are released in this case from the surface.

Once the antifouling character of MCP-6 was demonstrated, we evaluated its ability to provide efficient immobilization of biological probes. In particular, the immobilization rate of a panel of biological molecules including oligonucleotides, streptavidin, and antibodies was assessed.

The immobilization density of the different biological probes was measured using an indirect assay. The beads functionalized with capture probes were incubated with their biological counterparts. The different concentration of the target in the solution, before and after the incubation, was measured to assess the amount of analyte captured on the surface. This value allows inferring the quantity of the immobilized probe. This indirect assay could potentially underestimate the amount of probe that is bound to the surface. However, it reports the amount of probe that, most likely, takes part in the target recognition.

**Figure 4:**
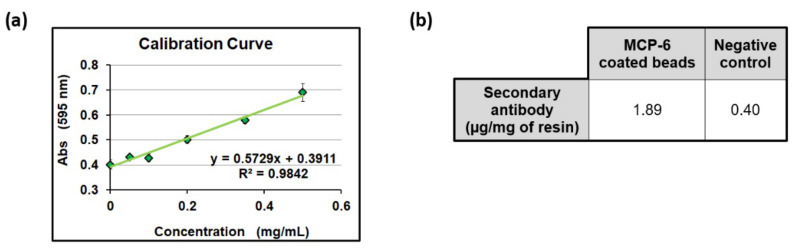
Antibody quantification by Bradford protein assay. (**a**) Calibration curve for goat antirabbit IgG; (**b**) concentration of goat antirabbit IgG captured on rabbit IgG-functionalized silica beads. The amount of captured IgG was calculated subtracting its concentration after the experiment with its initial concentration.

We first evaluated the ability of MCP-6-coated silica microbeads to immobilize DBCO-modified antibodies. A polyclonal rabbit IgG was functionalized with DBCO groups and incubated overnight with the beads to promote efficient immobilization. The negative control was obtained by incubating the beads overnight with just PBS. After several washing steps, both sets of beads were incubated with a secondary antibody (goat antirabbit IgG). The secondary antibody concentration was evaluated with a Bradford protein assay (see Figure 4) before and after the incubation. The density of the secondary antibody captured on the surface was 1.89 μg/mg of resin, while a sensibly lower density was found on the negative control (0.40 μg/mg of resin, around five times lower).

**Figure 5:**
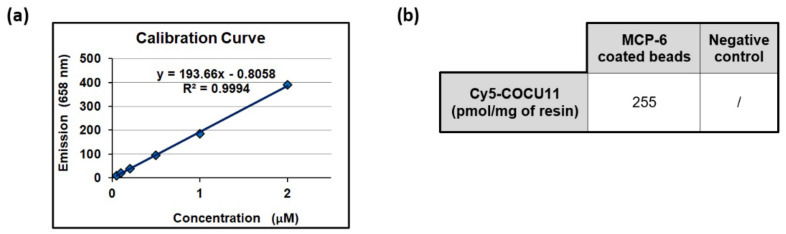
Spectrofluorimetric measurement of Cy5-labeled COCU11. (**a**) Calibration curve for Cy5-COCU11; (**b**) amount of pmoles of Cy5-COCU11 captured per mg of silica microbeads on the positive and negative control.

Similarly, we evaluated the immobilization of streptavidin on MCP-6-coated silica microbeads. DBCO-modified streptavidin, synthesized as described in Section 2.7.1, was incubated overnight with MCP-6-coated silica beads. The streptavidin immobilization yield was assessed by measuring the amount of biotinylated ssDNA (COCU8) that was effectively captured on the bead surface. As a negative control, a second set of beads was functionalized with biotin-labeled DNA-negative. Both sets of beads were incubated with Cy5-labeled COCU11 (a sequence of DNA complementary only to COCU8), and the fluorescence depletion of the solution was measured with a spectrofluorometer (see Figure 5). The results indicate that 255 pmoles per mg of resin was immobilized through hybridation with COCU8, while no COCU11 was captured on the negative control.

This result is important since it demonstrates that biotinylated DNA is specifically captured by streptavidin bound to MCP-6-coated silica beads. Additionally, the binding capacity of biotinylated ssDNA is slightly higher than that of commercially available beads (e.g., the reported binding capacity for Dynabeads™ Streptavidin M-270 is 200 pmol per mg of beads).

**Figure 6:**
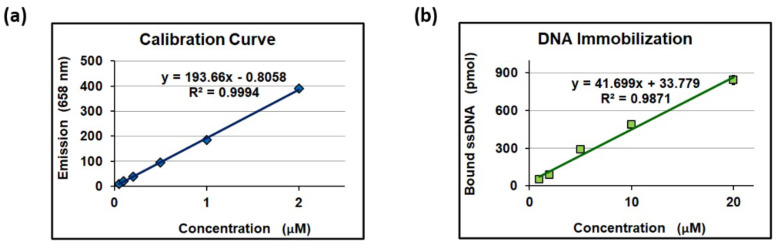
(**a**) Calibration curve for Cy5-labeled COCU11; (**b**) correlation between pmoles of ssDNA immobilized on 5 mg of MCP-6 coated beads and ssDNA concentration used.

Finally, the capacity of the binding ssDNA was evaluated. For this purpose, 5 mg of MCP-6 coated beads was incubated overnight with 100 μL of DBCO-modified COCU8 at different concentrations (namely 1, 2, 5, 10, and 20 μM) at 37 °C. After washing, the beads were incubated with 100 μL of Cy5-labeled COCU11 (at the same concentration used for COCU8 immobilization) for 1 h at 25 °C. After incubation, the supernatant was recovered, properly diluted, and the fluorescence emission evaluated with a spectrofluorometer. The COCU11 concentration was inferred using a calibration curve. The measured concentration was subtracted to the starting concentration to assess the amount of COCU11 captured on functionalized beads, and this value was used as an indirect measure of the COCU8 bound to MCP-6 beads. The results are shown in Figure 6.

As can be noticed, there is a linear correlation between the concentration of DBCO-modified COCU8 used for the immobilization on MCP-6 coated beads and the amount of ssDNA effectively immobilized on the surface. Since the molecular weight of DBCO-modified COCU8 and the total surface of 5 mg silica beads are known (8000 g/mol and 3.75 × 10^9^ μm^2^, respectively), the density of the DNA probes on the surface can be easily calculated and is reported in Table 1.

## 4. Conclusions

An effective strategy for the coating of silica microbeads was developed. MCP-6 was chosen to provide the functional coating that exposes the azido groups. Coating with MCP-6 gives excellent antifouling properties to silica particles, which are of utmost importance for their application in biological assays. Different biological probes have been successfully labeled with DBCO groups and immobilized onto coated silica particles, exploiting SPAAC reactions. Antibodies, streptavidin, and ssDNA have been used to functionalize silica beads and proved to still be able to interact with their biological counterparts (secondary antibody, biotinylated ssDNA, and complementary DNA, respectively).

## Figures and Tables

**Table 1:** Correlation between concentration and density of immobilized ssDNA on the surface.

Concentration * **(µM)**	1	2	5	10	20
Density ** **(ng/mm^2^)**	0.11	0.18	0.61	1.04	1.80

* Concentration used during incubation to immobilize COCU8 on the surface. ** Measured using the following equation: Density (ng/mm^2^) = (bound nmoles) × [molecular weight (g/mol)/total surface (mm^2^)].

## Data Availability

Not applicable.
